# DeepSimulator1.5: a more powerful, quicker and lighter simulator for Nanopore sequencing

**DOI:** 10.1093/bioinformatics/btz963

**Published:** 2020-01-08

**Authors:** Yu Li, Sheng Wang, Chongwei Bi, Zhaowen Qiu, Mo Li, Xin Gao

**Affiliations:** 1 Computer, Electrical and Mathematical Sciences and Engineering (CEMSE) Division, Computational Bioscience Research Center (CBRC), King Abdullah University of Science and Technology (KAUST), Thuwal 23955-6900, Saudi Arabia; 2 Tencent AI lab, Shenzhen 518000, China; 3 Biological and Environmental Sciences and Engineering (BESE) Division, King Abdullah University of Science and Technology (KAUST), Thuwal 23955-6900, Saudi Arabia; 4 Institute of Information and Computer Engineering, Northeast Forestry University, Harbin 150040, China

## Abstract

**Motivation:**

Nanopore sequencing is one of the leading third-generation sequencing technologies. A number of computational tools have been developed to facilitate the processing and analysis of the Nanopore data. Previously, we have developed DeepSimulator1.0 (DS1.0), which is the first simulator for Nanopore sequencing to produce both the raw electrical signals and the reads. However, although DS1.0 can produce high-quality reads, for some sequences, the divergence between the simulated raw signals and the real signals can be large. Furthermore, the Nanopore sequencing technology has evolved greatly since DS1.0 was released. It is thus necessary to update DS1.0 to accommodate those changes.

**Results:**

We propose DeepSimulator1.5 (DS1.5), all three modules of which have been updated substantially from DS1.0. As for the sequence generator, we updated the sample read length distribution to reflect the newest real reads’ features. In terms of the signal generator, which is the core of DeepSimulator, we added one more pore model, the context-independent pore model, which is much faster than the previous context-dependent one. Furthermore, to make the generated signals more similar to the real ones, we added a low-pass filter to post-process the pore model signals. Regarding the basecaller, we added the support for the newest official basecaller, Guppy, which can support both GPU and CPU. In addition, multiple optimizations, related to multiprocessing control, memory and storage management, have been implemented to make DS1.5 a much more amenable and lighter simulator than DS1.0.

**Availability and implementation:**

The main program and the data are available at https://github.com/lykaust15/DeepSimulator.

**Supplementary information:**

Supplementary data are available at *Bioinformatics* online.

## 1 Introduction

**Fig. 1.**
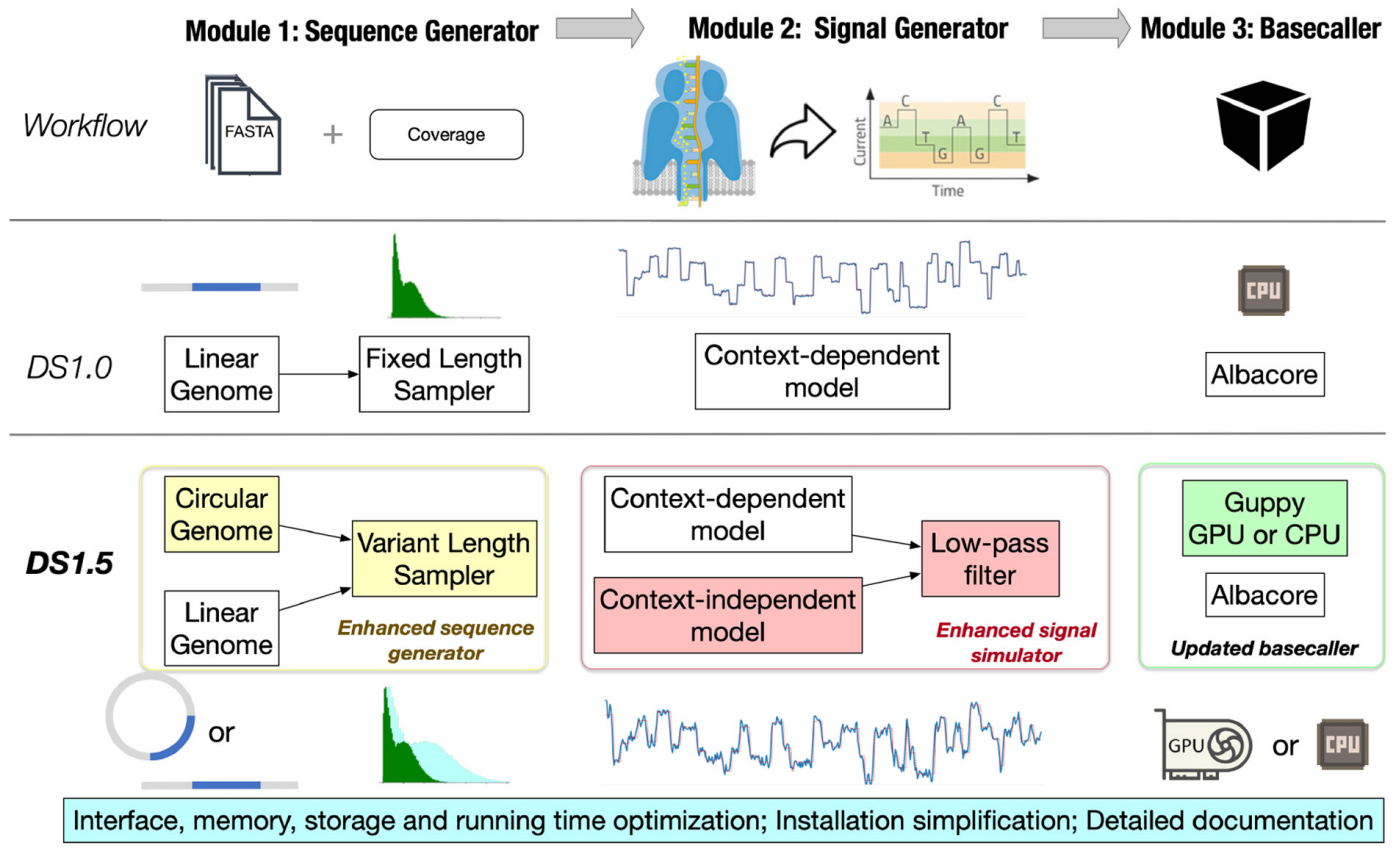
The overall workflow of DeepSimulator as well as the differences between DS1.5 and DS1.0. In brief, the DeepSimulator framework contains three modules: sequence generator, signal generator and basecaller. As shown in the last two rows, DS1.5 is significantly improved from DS1.0, with a greatly enhanced sequence generator and signal simulator, multiple new components as well as numerous optimizations. More discussions about the improvement of DS1.5 over DS1.0 can be referred to Section 2, Supplementary Sections S4 and S5

Because of its creative design and distinctive properties, i.e. portability, polymerase chain reaction-freeness and ultra-long reads, the Nanopore sequencing technology, which recognizes the nucleotides by detecting the electrical current signal changes when DNA or RNA molecules are forced to pass through a molecular pore (Li *et al.*, 2018), has achieved great success in recent years (Loman and Watson, 2015; Mueller *et al.*, 2019). Despite its clear advantages, Nanopore sequencing poses a number of computational challenges, for which various methods and algorithms have been developed (Han *et al.*, 2018; Senol Cali *et al.*, 2019; Wang *et al.*, 2018). Among them, simulators are an important type of tools (Baker *et al.*, 2016; Li *et al.*, 2018; Rohrandt *et al.*, 2018; Yang *et al.*, 2017; Yue and Liti, 2019). DeepSimulator (DS) (Li *et al.*, 2018), which we previously developed, was designed to simulate the Nanopore sequencing technology ‘deeply’, not only from the overall design aspect but also from the concrete algorithm aspect. Regarding the overall design (Fig. 1), we used three modules to mimic the real experimental procedures, which enable the simulator to simulate both the raw electrical current signals and the reads. As for the ‘deep’ algorithms, we deployed a specific deep learning model (Lam *et al.*, 2019; Li *et al.*, 2019), bi-directional long short-term memory (Bi-LSTM), which can capture both local and context information of the input sequences, to model the relation between the input sequences and the corresponding raw signals. Such designs can incorporate the error profile into the simulated signals and reads implicitly, which has been proved to benefit the simulation performance greatly (Li *et al.*, 2018).

Although the first version of DeepSimulator (DS1.0) has been recognized and used by a number of users (https://github.com/lykaust15/DeepSimulator) (Yue and Liti, 2019), there is still a large room for improvement. For example, though the final simulated reads have almost the same error distribution as the real reads, for some sequences, the divergence between the simulated raw signals and the real signals can be large, which can be inconvenient for the users who care about the signal outputs. In addition, the Nanopore technology has evolved greatly since DS1.0 was released. It is thus necessary to update DS significantly to accommodate those changes, such as the extended reads’ length. Here, we present a substantially updated version of DS, DeepSimulator1.5 (DS1.5), which is more powerful, quicker and lighter than DS1.0. In this new version, we have updated all the three modules substantially. Regarding the sequence generator, we updated the sample read length distribution to reflect the newest real reads’ features. In terms of the signal generator, which is the core of DS, we added one more pore model, the context-independent pore model, which is much faster than the previous context-dependent pore model. Furthermore, to make the generated signals more similar to the real ones and to make the simulator flexible enough to simulate signals with variant qualities reflecting the real-world complex situations, we added a low-pass filter to post-process the pore model signals. As for the basecaller, we added the support for the newest state-of-the-art basecaller, Guppy. Unlike Albacore, Guppy can support both GPU and CPU. In addition, multiple optimizations, related to multiprocessing control, memory and storage management, have been implemented to make DS1.5 a much more amenable and lighter simulator than the original DS1.0.

## 2 Workflow and implementation

Here, we summarize the main workflow of DS1.5. The simplified comparison between DS1.5 and DS1.0 can be found in Figure 1.

### 2.1 Sequence generator

DS was designed to simulate the entire Nanopore sequencing procedure, including sequence generator, raw signal generator and basecaller. Given the target genome sequence, the sequence generator samples sequences from the genome, which correspond to the DNA segments that pass through the molecular pore in the real experiments. Although this module is conceptually simple, we have included the following updates into DS1.5 to meet the needs of different users. Previously, by default, this module can only sample the linear genome. Now, we equipped it with the power to sample the circular genome or generate the reads without sampling. Furthermore, based on the feedback of the users (https://github.com/lykaust15/DeepSimulator/issues/13), we have made it easier to control the number of sampled reads and the coverage. Moreover, although the three read length distributions discussed in Li *et al.* (2018) have covered most of the circumstances in Nanopore sequencing, the overall average read length has become longer since the release of DS1.0. To accommodate this (https://github.com/lykaust15/DeepSimulator/issues/21), we have added an option for the users to specify the desired mean read length with the distribution still fitting the real case. More detailed discussion about this module can be referred to Supplementary Section S3.1.

### 2.2 Signal generator

The sampled sequences will go through the signal generator to output the simulated signals, whose behavior mimics that of a Nanopore sequencing device. In the signal generator, we use a deep learning-based pore model to produce the expected signals at each position of the input sequences. Then, each signal will be repeated several times based on the pattern in the real signals to produce the simulated signals (Supplementary Section Section S3.2). In DS1.0, we stopped at this step, which can be problematic because the output signals of this step consist of a series of square waves. To more realistically simulate the real-world Nanopore raw signals, we need to filter those high-frequency components embedded in the square waves. In DS1.5, we use a low-pass filter to achieve that, whose details can be found in the Supplementary Section S3.4. The low-pass filter and the thereafter Gaussian noise (Supplementary Section S3.5) can be used to control the quality of the output signals effectively. In addition to the re-designed signal processing pipeline, we have also updated the pore model, which is of crucial importance to DS. Previously, we implemented that with a Bi-LSTM model, resulting in a context-dependent pore model. That model works well under most circumstances. However, its computational requirements limit its application in large-scale simulations and metagenomic simulations. To overcome that limitation, we added a context-independent pore model (Supplementary Section Section S3.3) into DS1.5, which is based on the official statistics of 6-mers released by Nanopore Tech. The context-independent pore model is about 50 times faster than the context-dependent pore model, with acceptable accuracy compromise. Such an acceleration can broaden the application of DS1.5 greatly. We also preserved the context-dependent pore model and gave the user the freedom to switch between the two. Overall, DS1.5’s performance regarding simulating raw signals has been improved greatly from DS1.0. More details, including a continuous wavelet transformation analysis (Han *et al.*, 2018, 2019) on the simulated signals, can be referred to Supplementary Section S5.

### 2.3 Basecaller

After obtaining the signals produced by the signal generator, the next step is to translate the signals into the final reads, which correspond to the final sequence outputs in the real experiment. Although the users can feed a customized basecaller to DS, based on our experience, the users tend to use the default basecaller. Previously, the default basecaller of DS1.0 is Albacore. In London Calling 2019 (LC19), the Nanopore Tech has officially released a more powerful basecaller, Guppy. To cope with this evolution, we added both the GPU and CPU versions of Guppy into DS1.5 and made the GPU one the default basecaller (https://github.com/lykaust15/DeepSimulator/issues/20). At the same time, we preserved the option to use Albacore, in case the users need to do so.

### 2.4 Overall optimization

In addition to the aforementioned core updates, which are mainly made to improve the simulation quality, we have performed the following updates to improve the user experience. Firstly, we simplified the installation process: with only one command and no more configurations, the entire installation can be done. Secondly, we added threading management so that the users can control the resources allocated to the simulator. Thirdly, memory and storage management are optimized. The execution of DS1.5 is much lighter than that of DS1.0. On the other hand, all the intermediate results can still be outputted with optional parameters specified, if the users are interested in investigating them. Fourthly, we have refined the user interface as well as the overall code structure to make the code more readable so that the users can extend the tool or develop customized tools based on it. In addition, to help the users get used to DS with minimum efforts, we have provided multiple case studies with code in Supplementary Section S6 and the code repository of DS1.5 on Github.

## 3 Performances

From the user’s perspective, they can find three major improvements of DS1.5 regarding the performance. First of all, DS1.5 is much faster than DS1.0. The overall optimization and the context-independent pore model have sped up for a typical run 50 times with little simulation quality compromise, which allows the users to do large-scale read simulations. Secondly, with the help of the low-pass filter, the simulated signals from the enhanced signal simulator can mimic the real-world signals much better than those from DS1.0. Detailed results and comparisons can be found in Supplementary Section S3. Thirdly, because of the multiple updates in DS1.5, the profile of the simulated reads from DS1.5 can keep up with that of the real reads generated from the newest Nanopore chemistry.

## 4 Conclusions and discussion

In this work, we reported a new version of the previously published work on simulating the Nanopore sequencing, DeepSimulator1.5. In this updated version, we have updated all the three modules of DeepSimulator significantly with several crucial overall optimizations, resulting in a more powerful, quicker and lighter simulator. This major update can remarkably broaden its applications in large-scale sequencing simulations as well as studies focusing on the Nanopore signals. In the future, we will further equip DeepSimulator with the capability to simulate RNA sequencing and DNA modifications (Liu *et al.*, 2019; Xiao *et al.*, 2018; Ye *et al.*, 2016).

## Supplementary Material

btz963_Supplementary_Data
