# Supplementary material for: DeepSimulator1.5: a more powerful, quicker and lighter simulator for Nanopore sequencing
Source: Bioinformatics. 2020 Jan 8;36(8):2578–80. doi: 10.1093/bioinformatics/btz963 (PMC7178411; doi:10.1093/bioinformatics/btz963)
Supplement: btz963_Supplementary_Data [file btz963_supplementary_data.pdf]

Supplemental Materials for DeepSimulator1.5: a more powerful, quicker and lighter simulator for Nanopore sequencing

Yu Li[1,#], Sheng Wang[1,2,#,*], Chongwei Bi[3], Zhaowen Qiu[4], Mo Li[3], Xin Gao[1,*]

[1] King Abdullah University of Science and Technology (KAUST), Computational Bioscience Research Center (CBRC), Computer, Electrical and Mathematical Sciences and Engineering (CEMSE) Division, Thuwal, 23955-6900, Saudi Arabia.

[2] Tencent AI lab, Shenzheng, China

[3] King Abdullah University of Science and Technology (KAUST), Biological and Environmental Sciences and Engineering (BESE) Division, Thuwal, 23955-6900, Saudi Arabia.

[4] Institute of Information and Computer Engineering, NorthEast Forestry University, Harbin, China

[#] Contribute equally.

[*] All correspondense should be addressed to Xin Gao (xin.gao@kaust.edu.sa) and Sheng Wang (sheng.wang@kaust.edu.sa)

Contents

S1 Introduction and big picture of DeepSimulator v1.5 (DS1.5) 2

S2 Basic workflow of the signal simulator in DS1.5..................................... 3

S3 Detailed steps of the signal simulator in DS1.5 4

S4 Comparison between DS1.5 and DS1.0.. 14

S5 Detailed comparison on the signal level.. 16

S5 Useful examples 19

S6 Parameter explanation of DS1.5 21

Acknowledgments.. 22

References 22

S1 Introduction and big picture of DeepSimulator v1.5 (DS1.5)

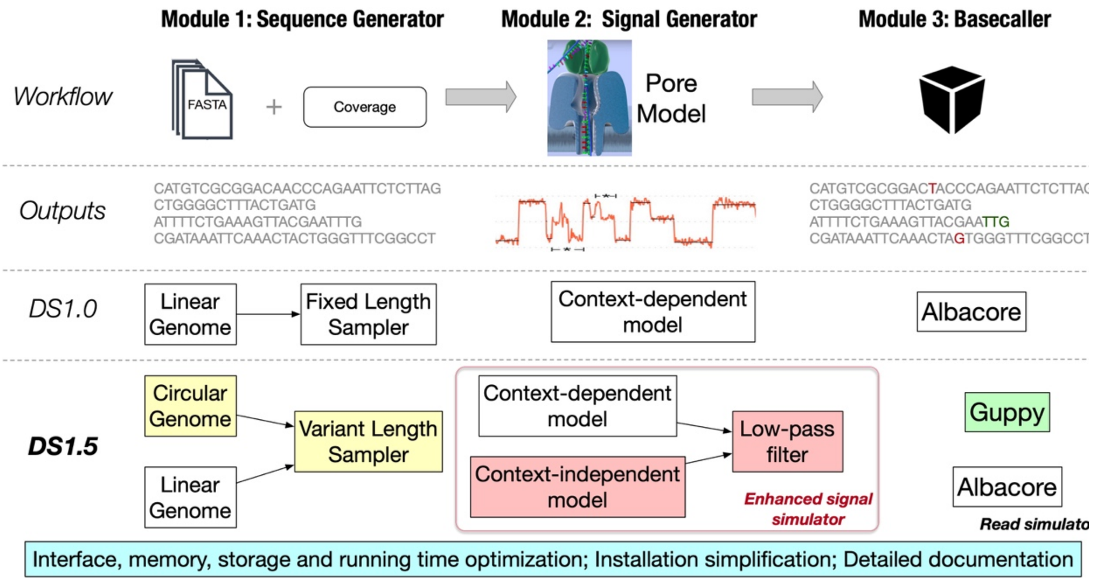

Supplementary Figure S1. The general workflow of DeepSimulator (DS), as well as a simplified comparison between DS1.5 and DS1.0. DS contains three modules: sequence generator, signal generator and basecaller. In DS1.5, the signal generator has been significantly enhanced. Now, combining sequence generator and signal generator, we can have the enhanced signal simulator, which can produce the simulated signals of much higher quality than that from DS1.0.

In Figure S1, we show the main workflow of DS as well as the difference between DS1.5 and DS1.0. In brief, DS contains three modules: sequence generator, signal generator and basecaller. The sequence generator is responsible for sampling reads from the reference genome. The signal generator can produce the expected signals given the input reads, which mimics the behavior of the real Nanopore devices. The basecaller is used to produce the simulated reads from the simulated signals. In DS1.0, while its function of simulating reads has been satisfactory, its ability of simulating signals is limited. In DS1.5, we significantly enhanced the signal simulating components in the DS framework. In Section S2 and Section S3, we introduce the enhanced sequence generator and the signal simulator in DS1.5 in detail. On the other hand, combining the signal simulator and the basecaller, we can have a read simulator. With an enhanced signal simulator, our read simulator can also be improved. In Section S4, we give a summary table to show the detailed improvements of DS1.5 from DS1.0, and, in section S5, we give a more detailed comparison between those two versions on the signal level. Section S6 will give some examples of using DS1.5 with the parameters given. The last section, Section S7, gives a summary of all the available parameters of DS1.5.

S2 Basic workflow of the signal simulator in DS1.5

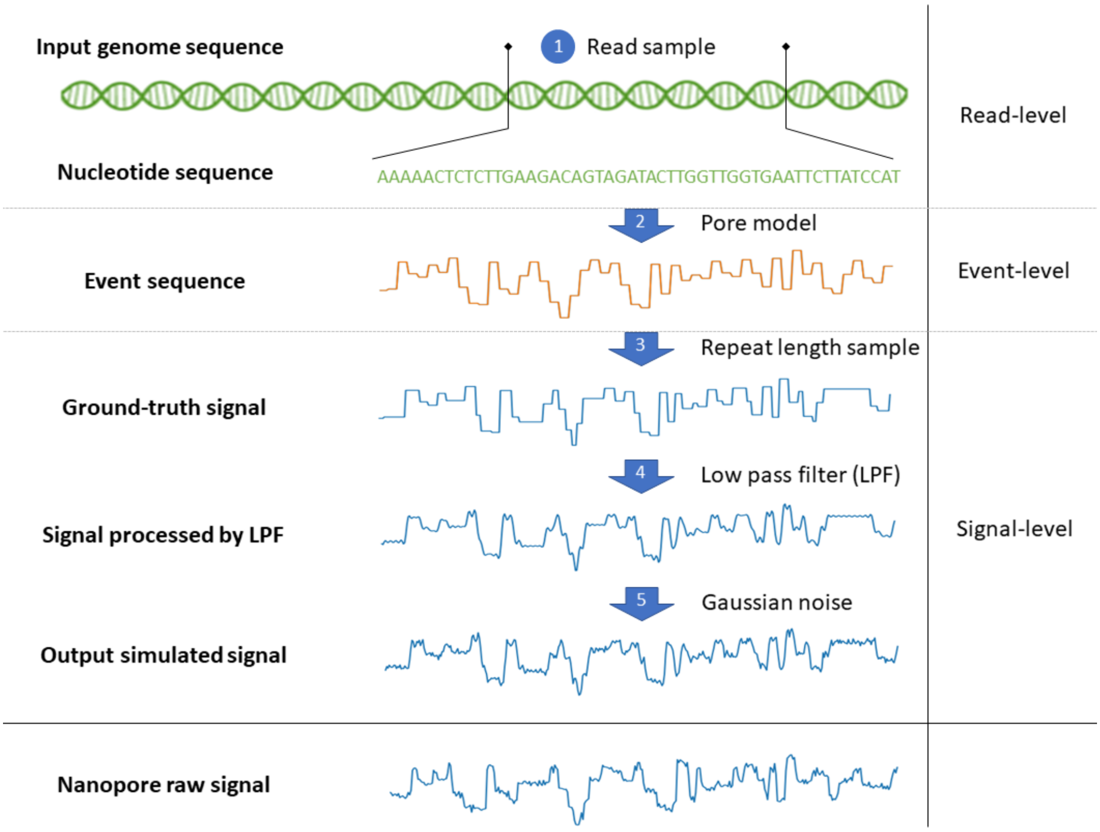

Supplementary Figure S2. Illustration of the workflow of the first two modules in DS1.5 with five components. (1) Given an input genome sequence, DS1.5 randomly chooses starting positions on the genome to produce the sampled nucleotide sequences; (2) then DS1.5 will generate the event sequences from the nucleotide sequences by the pore model [1]; (3) later, the event sequences are used to produce the ground-truth signals according to the repeat length distribution. Finally, to simulate the real-world Nanopore raw signals, DS1.5 applies low pass filter (4) and adds the Gaussian noise (5) on the ground-truth signals. It is shown that the output simulated signals highly resemble the Nanopore raw signals.

S3 Detailed steps of the signal simulator in DS1.5

S3.1 Nucleotide sequences from the input genome

Starting from the input genome, the users may specify either the coverage or the number of reads to produce the sampled nucleotide sequences. In particular, DS1.5 randomly chooses a starting position on the genome to produce the sampled nucleotide sequences, which satisfy the coverage requirement and the length distribution of the experimental Nanopore reads.

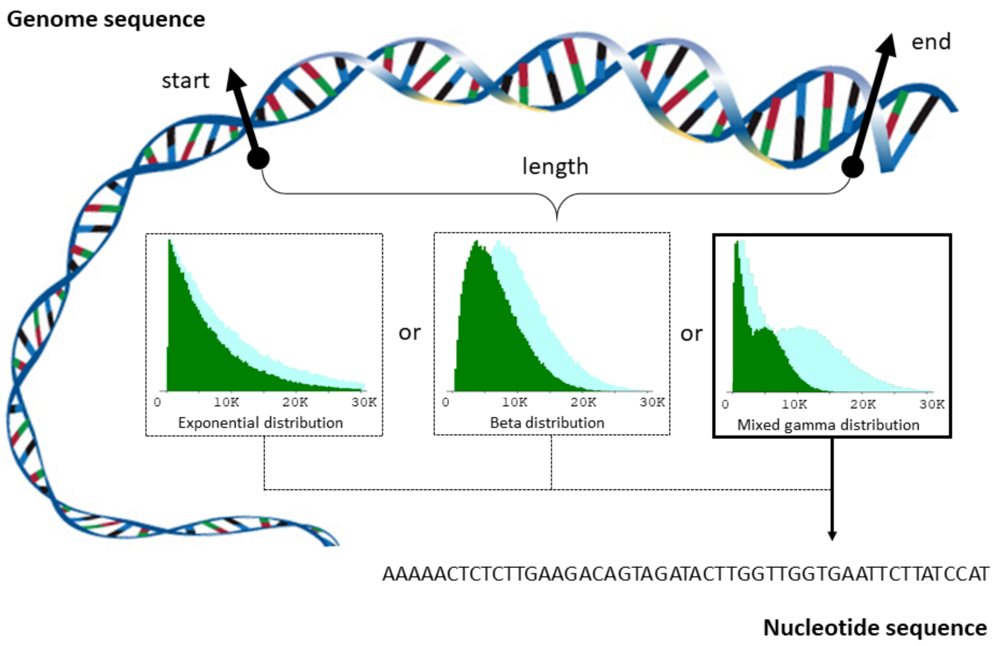

Supplementary Figure S3. The read sampling component of DS1.5. From the input genome sequence, DS1.5 first randomly chooses starting positions on the genome, and then samples read lengths from a user-specified distribution to produce the nucleotide sequences. Three length distributions are provided and the mixed gamma distribution is set as default. In addition, the users can also tune the distributions by the mean value according to their purposes, as shown in different colors (green or cyan) in this figure.

As discussed previously [1,2], many factors, such as the experimental purpose and the user's experience, would influence the read length distribution greatly. To fulfill these needs, we provide three patterns for the length distribution through the investigation of a variety of Nanopore sequencing datasets [1]: (a) exponential distribution, (b) beta distribution, and (c) mixed gamma distribution (shown in Figure S3). By default, DS1.5 will choose the mixed gamma distribution to sample the length of the nucleotide sequence, and the mean value of the length is set as 8K. However, the users can also tune this mean value to any other numbers according to their purposes (shown in Figure S3).

S3.2 Event sequence from the pore model

The pore model in Nanopore sequencing is used to model the expected current signal of a given k-mer (k equals to 6 for the R9.4 pore chemistry) nucleotides from the single strand DNA that passes through the pore [3].

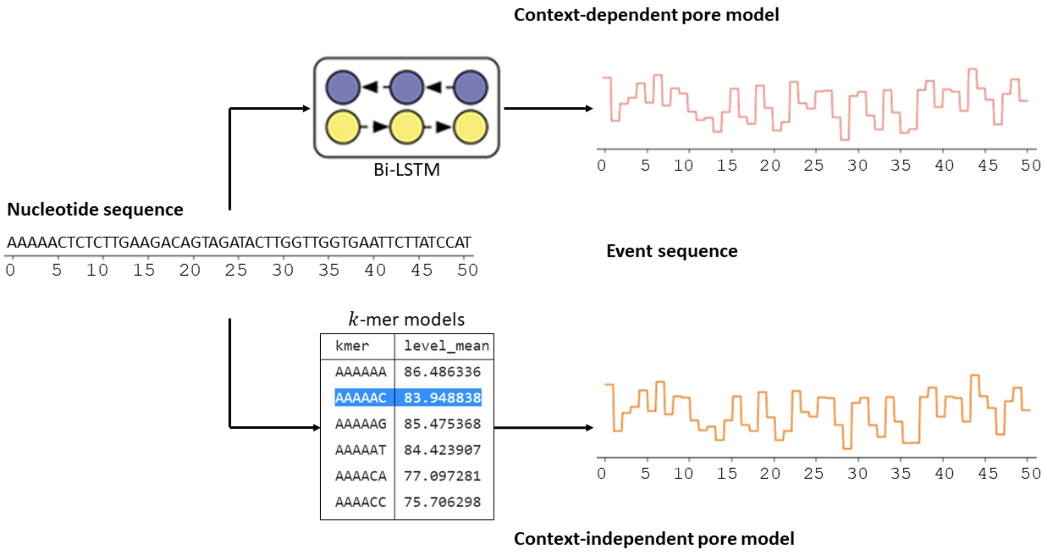

Supplementary Figure S4. The pore model component of DS1.5. From the nucleotide sequences, DS1.5 will generate the event sequences (i.e., the expected current signal of a 6-mer nucleotides from the nucleotide sequences) from either the context-dependent pore model or the context-independent pore model. The context-dependent pore model is a Bi-LSTM (bi-directional Long Short-Term Memory) neural network [1] which can generate the event sequences considering its context in the nucleotide sequences. Conversely, the context-independent pore model simply applies the 6-mer model [4] to generate the event sequences.

Formally, this problem could be formulated as follows: given an input nucleotide sequence $X = x_1, x_2, \dots, x_L$ with L nucleotides where x_i is a 4-state nucleotide base that can take one of the four values from {A,T,C,G} for DNA, we need to generate the corresponding expected electrical current signals $Y = y_1, y_2, \dots, y_{L-5}$ where y_i is the expected current signal of a 6-mer starting from position i in X (e, g, 'ACCGTT'). To simplify the problem, we elongate the length of Y to the same length as X by padding the end of Y with either the last value of Y (i.e., y_{L-5}) or the average expected signal value of all 6-mers. We denote this generated Y sequence as the event sequence.

DS1.5 allows the users to choose from two pore models: the context-independent and the context-dependent pore model. Regarding the context-independent pore model, each 6-mer from the nucleotide sequence is assigned with a fixed value as the expected current signal [4] regardless of its location on the nucleotide sequence (the lower part of Figure S4); on the

other hand, the context-dependent pore model makes use of a deep learning model, i.e., Bi-LSTM [1] to assign the expected current signal values for each 6-mer, which might be different for the same 6-mers locating in different places on the DNA sequence because this pore model also considers the context of each 6-mer in the nucleotide sequence (the upper part of Figure S4).

S3.3 Ground-truth signal

With the current MinION pore chemistry, Oxford Nanopore Technologies (ONT) reports that single DNA strands are pulled through the pore at an average speed of 450 bp/s, while the electric current is sampled at a frequency of 4kHz. Consequently, there are on average eight to nine discrete measurements per k-mer event [5].

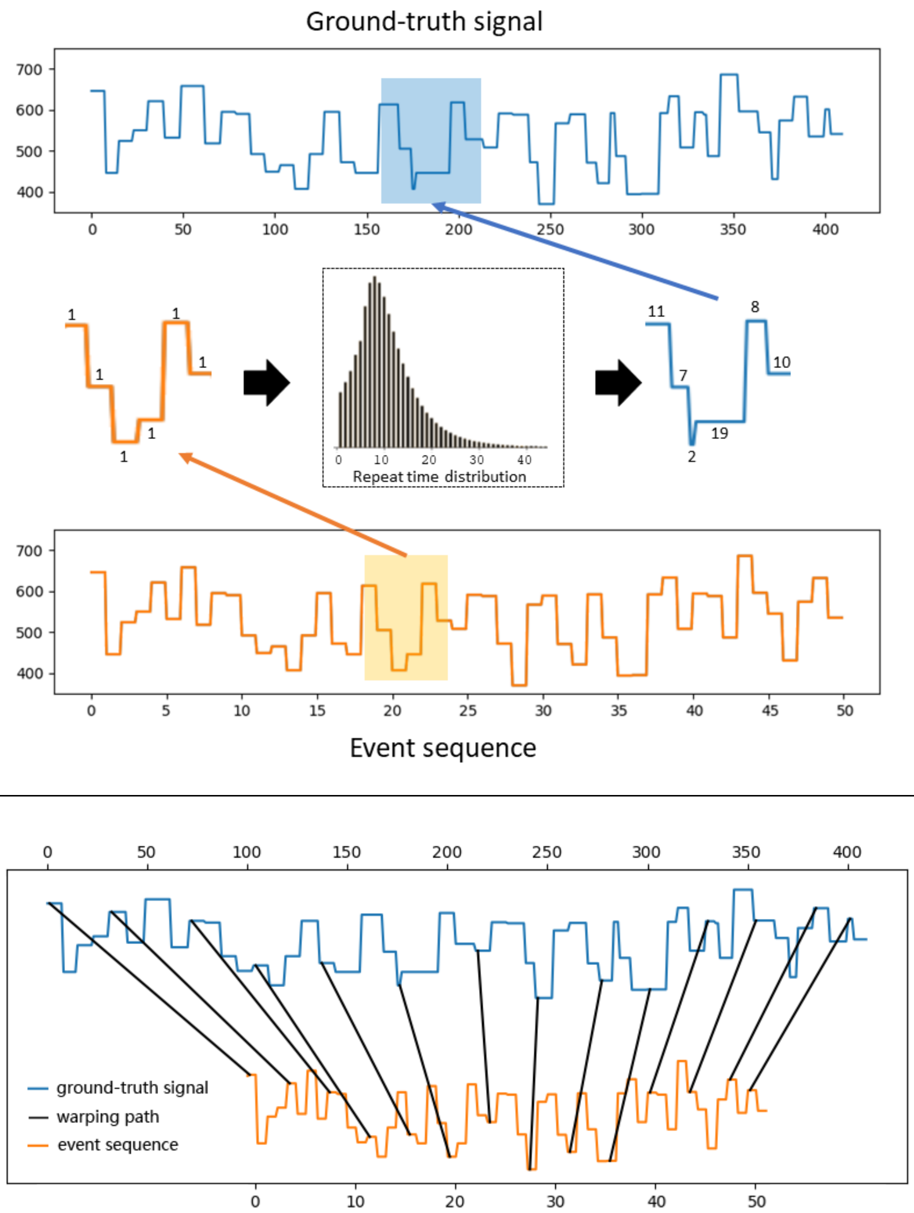

Supplementary Figure S5. The repeat length sampling component of DS1.5. From the event sequence, DS1.5 samples the repeat time for each event to produce the ground-truth signal (i.e., the simulated signal without any randomization), which satisfies the MinION pore chemistry. Noticeably, during this process, the ground-truth warping path between the ground-truth signal and the event sequence can be recorded for downstream analysis.

To simulate this process, as shown in the upper part of Figure S5, we repeat N times for each k-mer from the event sequence where N is sampled from a mixture alpha distribution summarized from the statistics of a variety of Nanopore sequencing datasets [1]. Therefore, this procedure can generate the ground-truth signal (i.e., the simulated signal without any randomization) whose alignment (or, warping path) to the event sequence is recorded simultaneously (the lower part of Figure S5). Notice that it is difficult, if not impossible, to obtain the ground-truth warping path for the real Nanopore raw signals. Unlike the real dataset, whose ground-truth is usually lacking, the ground-truth warping path from DS1.5 might serve as the gold standard for testing the signal labeling algorithms [6] in Nanopore sequencing.

Now the length of the simulated ground-truth signals (about 8 to 9 times longer than the event sequence) is compatible to that of the raw signals as measured from Nanopore sequencing. When feeding these simulated ground-truth signals from the context-independent pore model to the base-callers, we can obtain >96% base-calling accuracy.

To generate the ground-truth signals as well as the alignments, please run DS1.5 with the parameters “-O 1 -P 1”.

S3.4 Low pass filter

The simulated ground-truth signals are composed of a series of square waves (the top in Figure S6), whose frequency spectra consist of an infinite series of sine wave harmonics. Therefore, to simulate the signals that resemble the real-world Nanopore raw signals, we need to filter those high-frequency components embedded in the square waves, which can be fulfilled with low-pass filters.

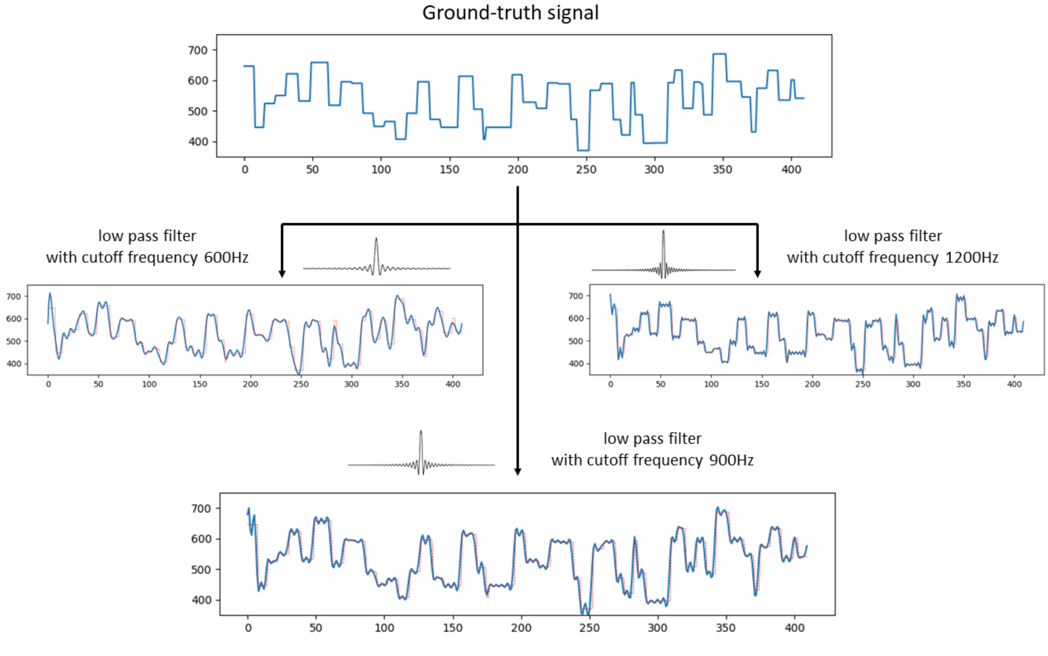

Supplementary Figure S6. The low-pass filter component of DS1.5. To mimic the real-world Nanopore sequencing process in which the sampling frequency is fixed as 4kHz, it is necessary to filter those high-frequency components embedded in the square waves of the ground-truth signals. This can be realized by the low-pass filter (LPF) with a given cutoff frequency that shall be larger than the frequency of the actual signal in Nanopore sequencing (say, 450Hz, which corresponds to the speed that single DNA strands are pulled through the pore at around 450 bp/s). With the increase of the cutoff frequency in LPF, the filtered signals will behave from "smoother" to "sharper" especially at the edge between two square waves of the ground-truth signal (shown in dotted red curves).

A low-pass filter (LPF) is a filter that passes signals with a frequency lower than a selected cutoff frequency and attenuates signals with frequencies higher than the cutoff frequency [7]. Practically, this can be realized by convolving the input signal with a windowed-sinc function. Three key parameters control an LPF: sampling frequency, cutoff frequency, and transition bandwidth. In our task, the sampling frequency is fixed to 4kHz; considering the maximal frequency of the actual signal in Nanopore sequencing (i.e., the speed that single DNA strands

are pulled through the pore at around 450 bp/s), the cutoff frequency should be larger than 450Hz and is the only tunable parameter for the windowed-sinc function; the transition bandwidth controls the width between the passband frequency and the cutoff frequency, and can be fixed to 40Hz in our task (i.e., about 10% to the frequency of the actual signal).

As shown in the lower part of Figure S6, different cutoff frequencies will produce different filtered signals from the input ground-truth signals in square waves. A lower cutoff frequency will filter out more high-frequency component in the square waves, which will generate "smoother" signals especially at the edge between two square waves. On the other hand, a higher cutoff frequency will produce "sharper" signals, and there are waveforms of small amplitude high-frequency vibration near the edges.

Experiments show that a higher cutoff frequency value would result in better base-calling accuracy and the lower value will reduce the base-calling accuracy. Please refer to Table S1 for the relationship between the cutoff frequency value and the base-calling accuracy, when canceling all the other randomness in DS1.5 (such as the Gaussian noise of the event and the signal) using the context-independent pore model. By default, we set the cutoff frequency as 950Hz, which doubles the average frequency of the actual signal.

Supplemental Table S1. The base-calling accuracies and the simulated signals corresponding to different cutoff frequencies. We also provide the relevant parameters of DS1.5 to produce these results.

Cutoff freq (in Hz)	Parameters of DS1.5	Base-calling accuracy	Simulated signals
600	-e 0 -f 600 -s 0	71.4%	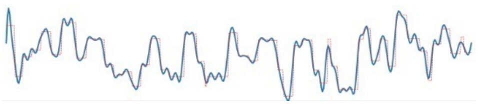
650	-e 0 -f 650 -s 0	72.9%	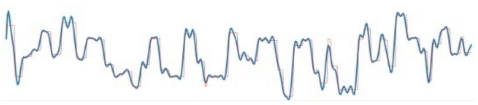
700	-e 0 -f 750 -s 0	77.0%	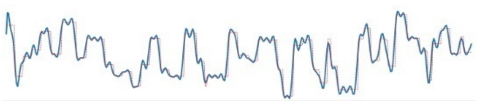
750	-e 0 -f 700 -s 0	82.6%	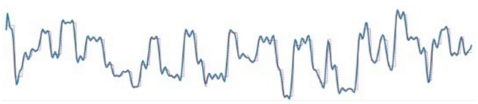
800	-e 0 -f 800 -s 0	87.7%	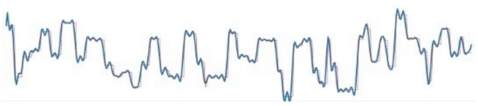
850	-e 0 -f 850 -s 0	91.0%	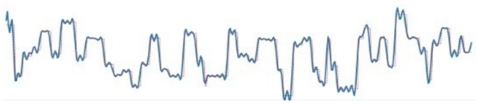

900	-e 0 -f 900 -s 0	93.0%	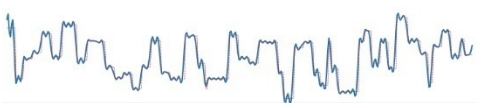
950	-e 0 -f 950 -s 0	94.2%	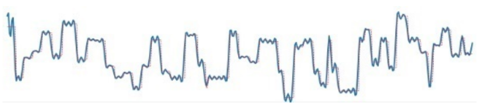
1000	-e 0 -f 1000 -s 0	95.0%	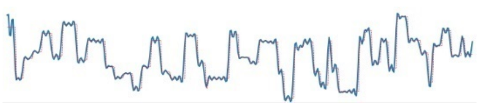
1050	-e 0 -f 1050 -s 0	95.6%	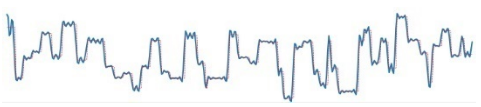
1100	-e 0 -f 1100 -s 0	96.0%	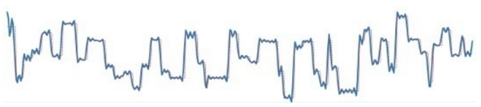
1150	-e 0 -f 1150 -s 0	96.3%	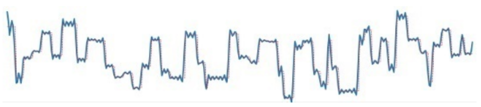
1200	-e 0 -f 1200 -s 0	96.6%	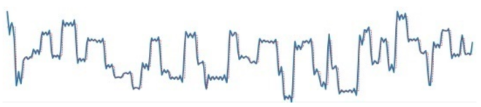

S3.5 Gaussian noise

It should be noted that the Nanopore raw signals are extremely noisy due to the complicated sequencing environment [3]. To simulate this effect, we add Gaussian noise with the user-defined variance parameter to each position of the simulated signals. As shown in Table S2, if the standard deviation of the Gaussian noise at the signal-level is set to a smaller value, the simulated signal will be smoother, resulting in a higher base-calling accuracy; conversely, a larger Gaussian noise will produce a fuzzier simulated signal, which reduces the base-calling accuracy. We suggest the users to choose the parameter "-s" from 1.0 to 2.0, which will generate the simulated signals more like the raw signals.

Supplemental Table S2. The relation between the Gaussian noise at the signal-level and the base-calling accuracy as well as the simulated signals. We also provide the relevant parameters of DS1.5 to produce these results.

Gaussian noise at signal-level	Parameters of DS1.5	Base-calling accuracy	Simulated signals
0.5	-e 0 -f 950 -s 0.5	94.2%	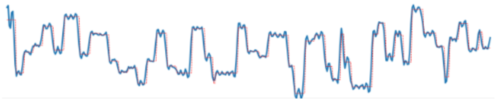
1.0	-e 0 -f 950 -s 1.0	93.8%	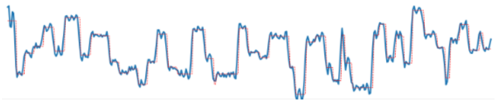
1.5	-e 0 -f 950 -s 1.5	92.7%	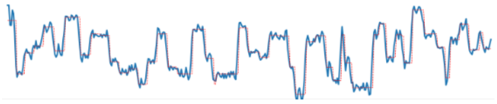
2.0	-e 0 -f 950 -s 2.0	90.9%	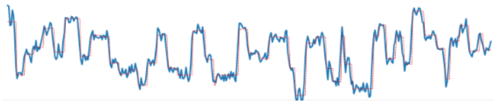
2.5	-e 0 -f 950 -s 2.5	86.9%	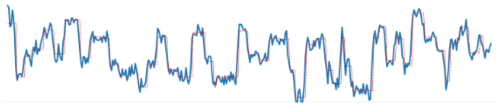
3.0	-e 0 -f 950 -s 3.0	78.4%	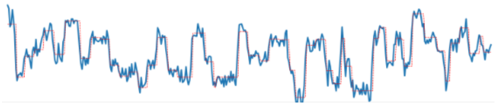

If the context-independent pore model is chosen, the users can set the standard deviation of the Gaussian noise at event-level. With this operation, the same 6-mer in different positions along the nucleotide sequence can have different expected current signal values. As shown in Table S3, if the standard deviation of the Gaussian noise at the event-level is set to a smaller value, the simulated signals will be closer to the ones generated by the original event

sequences, which leads to a higher base-calling accuracy; on the contrary, a larger Gaussian noise will output deviated signals, which deteriorates the base-calling accuracy. We recommend the users to choose the parameter "-e" from 0.5 to 1.5 for producing an effective randomness upon the original event sequences, whose effect will resemble the context-dependent pore model.

Supplemental Table S3. The relation between the Gaussian noise at the event-level and the base-calling accuracy as well as the simulated signals. We also provide the relevant parameters of DS1.5 to produce these results.

Gaussian noise at event-level	Parameters of DS1.5	Base-calling accuracy	Simulated signals
0.5	-e 0.5 -f 950 -s 0	94.0%	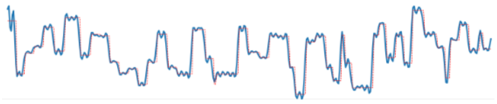
1.0	-e 1.0 -f 950 -s 0	92.9%	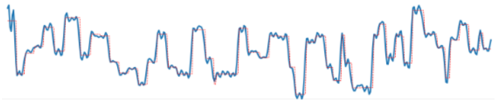
1.5	-e 1.5 -f 950 -s 0	90.3%	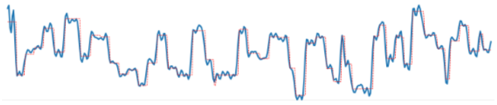
2.0	-e 2.0 -f 950 -s 0	85.5%	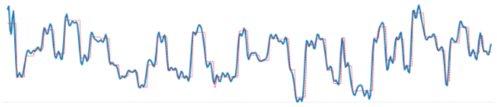
2.5	-e 2.5 -f 950 -s 0	79.6%	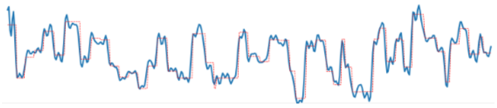
3.0	-e 3.0 -f 950 -s 0	75.0%	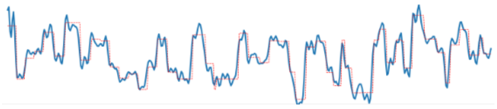

S4 Comparison between DS1.5 and DS1.0

Supplemental Table S4. Overview of the tool comparison between DS1.0 and DS1.5 on the read-level, the event-level, and the signal-level, respectively.

Tools	Read-level			Event-level			Signal-level		Basecaller
	Circular genome	Genome coverage	Variable read_len	Context-dependent	Context-independent	Event noise	LPF	Signal noise	Guppy support
DS1.0	×	×	×	✓	×	×	×	✓	×
DS1.5	✓	✓	✓	✓	✓	✓	✓	✓	✓

S3.1 Read-level

In DS1.5, users can determine the average length of the sampled read by specifying the parameter "-l <read_len>". We also allow the users to set the sequencing coverage, in consideration of both the average read length and the length of the input genome, with the parameter "-K <coverage>". This can automatically determine the number of reads to be simulated. If both coverage (i.e., parameter "-K") and read number (i.e., parameter "-n") are given, we use the larger one. Furthermore, we also provide the option to simulate the read on circular genome, which can be achieved by specifying "-C" as 1. The underlying reason is that the genomes from a variety of bacteria and mitochondria are circular and they are critical for both health and research. Within the software, we realize that by sampling the reads from the genome which is represented as a circular sequence.

S3.2 Event-level

DS1.5 allows the users to apply either the context-dependent or the context-independent pore model to generate the event sequence. By default, the context-independent pore model is chosen, as it runs 10-20 times faster than context-dependent pore model while produces almost the same event-level sequence. Measured by $nDist$ from Dynamic Time Warping (DTW) [6], the difference between the two event-level sequences generated by the two pore models is only around 0.06, which means that they are very similar. For the event sequence generated by the context-independent pore model, DS1.5 further allows the users to add Gaussian noise to make the simulation more like the actual process.

S3.3 Signal-level

DS1.5 employs low-pass filter (LPF) to remove those high-frequency components embedded

in the square waves of the ground-truth signal that is derived from the event sequence. This will make the simulated signals resemble the real-world Nanopore raw signals (as shown in Figure S2).

S3.4 Basecaller

Since London Calling 2019 (LC19), the best basecaller for Nanopore sequencing has changed from Albacore to Guppy. We reflect this change in DS1.5. Now, the default basecaller of DS1.5 is the GPU version of Guppy. Considering some groups are still using Albacore, we preserve the option of changing the basecaller back to Albacore. The users can achieve this by specifying the parameter "-B" as 2. If the users want to have a taste of Guppy's performance while do not want to install the CUDA kit, they can use the CPU version of Guppy by specifying the parameter "-B" as 1, although the CPU version is much slow than the GPU one.

S5 Detailed comparison on the signal level

In this section, we further provide a more detailed comparison between the signals generated by DS1.0 and DS1.5, with the ground truth raw signals.

To evaluate the divergence of the simulated signals from the corresponding raw signals, we first randomly sampled 100 raw signals and obtained the ground-truth reference reads from the NA12878_Human_Chr21 dataset. Then, those ground-truth reads were feed to DS1.0 and DS1.5 to generated the corresponding simulated signals, respectively. Since, in the DS framework, we introduce the randomization into the event repeat time, the simulated signals may not have the same length as the corresponding raw signals. For the sake of the downstream analysis and comparison, we used cwDTW [6] to map the simulated signals onto the corresponding raw signals and re-warped each signal, making it have the same length as the corresponding raw signal.

After the above preprocessing, we had 100 groups of signals. Each group is composed of one raw signal sequence, one simulated signal sequence from DS1.0, and one simulated signal sequence from DS1.5, of the same length. Next, we compared the two versions of simulated signals against the ground truth raw signals.

(A) The real raw signals

(B) Simulated signals from DS1.5

(C) Simulated signals from DS1.0

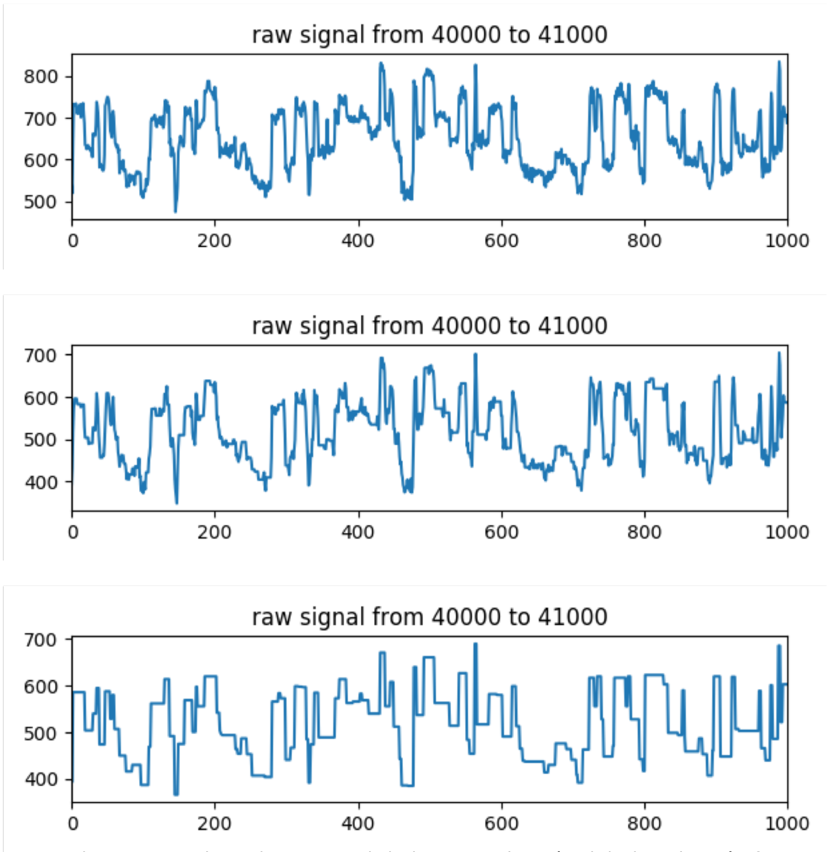

Supplementary Figure S7. The comparison between (A) the raw signals, (B) the signals from DS1.5, and (C) the signals from DS1.0. Compared to DS1.0, the signals from DS1.5 are more similar to the ground truth raw signal, containing more correct detailed information.

Firstly, we visualized one group of signals, whose results are shown in Figure S7. As shown in the figure, overall, both the signals from DS1.0 and DS1.5 are similar to the ground truth signal sequence, which suggests the effectiveness of the DS framework. On the other hand, most of the signals from DS1.0 form square waves, which means they contain too many wrong high-frequency details. DS1.5 resolves the problem precisely with a low-pass filter and attenuates the wrong high-frequency details in the signals, whose outputs are more similar to the ground truth signals visually, as illustrated in Figure S7 (A, B).

However, the preserved high-frequency details can be noisy and may not have any correlation with the ground truth, which can make the simulated signals even worse. To evaluate the high-frequency components in the DS1.5 signals, we further performed a continuous wavelet transformation analysis (CWT) on all the signal sequences. Essentially, for each signal sequence, we used a series of wavelets with different frequencies (or, scales) to perform 1D convolution on it. Since each wavelet at a given scale can generate a transformed feature signal sequence for an input signal sequence, the CWT analysis can generate a 2D matrix for each input signal sequence, which contains both the low-frequency transformation and the high-frequency transformation at each time point (i.e., time/frequency analysis). Therefore, such a 2D matrix is denoted as the CWT spectrum.

Figure S8 shows the CWT transformation results for a group of signals. Overall, the CWT spectra of DS1.0 signals and DS1.5 signals are similar to that of the real raw signals. However, in the high frequency part, DS1.5 is more similar to the real signals than DS1.0. We also measured the similarity of DS1.5 VS Raw and DS1.0 VS Raw in terms of the CWT spectrum quantitatively, which is reported in Table S5. Within each group, we calculated the L2 distance (Frobenius distance) between the DS 1.5 spectrum and the raw signals spectrum, as well as the correlation between the two spectra. We also calculated the correlation between the two for the high frequency (the top 10 components) in particular. The averaged values from the 100 groups are reported in the table. We further performed the same analysis for the comparison between DS1.0 spectra and ground truth spectra, summarizing the results in the

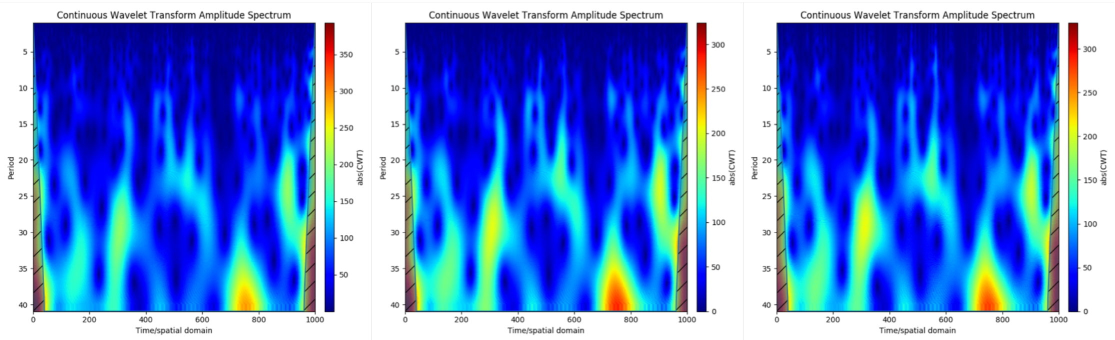

(A) Real signals (B) DS1.5 signals (C) DS1.0 signals

Supplementary Figure S8. The comparison between real raw signals, the signals from DS1.5, and the signals from DS1.0, with continuous wavelet transformation analysis (CWT). The CWT spectrum for DS1.5 (B) is more similar to the spectrum for the real signals (A) than that for the signals from DS1.0 (C). Specifically, we can find that the colored spot in the high-frequency part can overlap between the first two figures, which means that DS1.5 signals are very similar to the real signals. On the other hand, the difference between the real signals and the DS1.0 signals is not that large, which suggests the effectiveness of the DS framework.

From Table S5, we can find that the difference between DS1.5 spectrum and the raw spectrum is indeed much small than the difference between DS1.0 spectrum and the raw spectrum, which is improved by 21.35% in terms of L2 distance and by 1% in terms of Pearson correlation coefficient (PCC). Moreover, the improvement of PCC in the high-frequency part is even higher (2.48%), which suggests that the high-frequency details processed by the low-pass filters are indeed consistent with the ground truth signals and have improved the signal quality.

Supplemental Table S5. Quantitative comparison between the CWT spectra of different signals. We evaluate 100 groups of spectra and report the comparison here. The spectra of DS1.5 signals are more similar to those of the real raw signals than the DS1.0, especially on the high frequency.

	L2 distance	PCC (high frequency)	PCC (all)
Raw VS DS1.0	955.46	0.886	0.979
Raw VS DS1.5	751.44	0.908	0.988
Improvement	**21.35%**	**2.48%**	**1.00%**

S6 Useful examples

In this section, we provide several useful examples of DS1.5, ranging from the generation of the event sequence, to the simulation of the Nanopore sequencing at a given coverage.

1. The event sequence for a given sequence

1.1 The event sequence from the context-dependent pore model

./pore_model.sh example/001c577a-a502-43ef-926a-b883f94d157b.true_fasta 0

1.2 The event sequence from the context-independent pore model

./pore_model.sh example/001c577a-a502-43ef-926a-b883f94d157b.true_fasta 1

2. Generate the ground-truth signal for a given sequence

./deep_simulator.sh -i example/001c577a-a502-43ef-926a-b883f94d157b.true_fasta -n -1 -P 1

3. Simulate 100 reads/signals for a given genome

./deep_simulator.sh -i example/artificial_human_chr22.fasta -n 100

4. Simulate the reads at different base-calling accuracy

4.1 accuracy around 95%

./deep_simulator.sh -i example/artificial_human_chr22.fasta -e 0.5 -f 1100 -s 1.0

4.2 accuracy around 90%

./deep_simulator.sh -i example/artificial_human_chr22.fasta -e 0.5 -f 900 -s 1.5

4.3 accuracy around 85%

./deep_simulator.sh -i example/artificial_human_chr22.fasta -e 1.0 -f 850 -s 2.0

4.4 accuracy around 80%

./deep_simulator.sh -i example/artificial_human_chr22.fasta -e 1.5 -f 800 -s 2.0

5. Simulate the Nanopore sequencing at 20x coverage

./deep_simulator.sh -i example/artificial_human_chr22.fasta -K 20

6. Simulate the Nanopore sequencing with read length around 4000

./deep_simulator.sh -i example/artificial_human_chr22.fasta -l 4000

7. Simulate the Nanopore sequencing with the CPU version Guppy

./deep_simulator.sh -i example/artificial_human_chr22.fasta -B 2

S7 Parameter explanation of DS1.5

Supplemental Table S6. The main parameters of DeepSimulator v1.5.

Stage	Parameter	Explanation
Read-level	-n read_num	The number of reads need to be simulated.
	-C circular_genome	Specify whether the input is circular genome.
	-K coverage	The sequencing coverage, which will be converted to the number of reads in consideration of the average read length.<br>[note]: If both -K and -n are given, we use the larger one.
	-l read_len	The average read length to be sampled.
	-m read_len_distri	The length distribution used for the read sampling. 1: beta distribution, 2: exponential distribution, 3: mixed gamma distribution. Default is 3.
Event-level	-M pore_model	Choose context-dependent (0) or context-independent (1) pore model to generate the event sequence. Default is 1.
	-e event_std	Set the standard deviation of the random noise on the event sequence. Default is 1.0.
	-O out_align	Output the ground-truth warping path between the simulated signal and the event sequence.
Signal-level	-f filter_freq	Set the cutoff frequency for the low pass filter. Default is 950.
	-s signal_std	Set the standard deviation of the random noise on the generated signal. Default is 1.0.
	-P perfect	Generate the ground-truth signal with no randomization and low pass filter.
Basecaller	-B basecaller	Choose the basecaller for the basecalling process.

Acknowledgments

We would like to thank all the users that have discussed with us on Github, which have definitely improved DeepSimulator greatly, resulting in DeepSimulator1.5. We want to thank Jia-Xing Yue especially for his constructive feedbacks. The research reported in this paper was supported by funding from King Abdullah University of Science and Technology (KAUST), under award number FCC/1/1976-18-01, FCC/1/1976-23-01, FCC/1/1976-25-01, FCC/1/1976-26-01, and FCS/1/4102-02-01.

References

1. Li, Y., Han, R., Bi, C., Li, M., Wang, S., & Gao, X. (2018). DeepSimulator: a deep simulator for Nanopore sequencing. *Bioinformatics*, *34*(17), 2899-2908.
2. Yang, C., Chu, J., Warren, R. L., & Birol, I. (2017). NanoSim: nanopore sequence read simulator based on statistical characterization. *GigaScience*, *6*(4), gix010.
3. David, M., Dursi, L. J., Yao, D., Boutros, P. C., & Simpson, J. T. (2016). Nanocall: an open source basecaller for Oxford Nanopore sequencing data. *Bioinformatics*, *33*(1), 49-55.
4. K-mer models from ONT https://github.com/nanoporetech/kmer_models
5. Rang, F. J., Kloosterman, W. P., & de Ridder, J. (2018). From squiggle to basepair: computational approaches for improving nanopore sequencing read accuracy. *Genome biology*, *19*(1), 90.
6. Han, R., Li, Y., Gao, X., & Wang, S. (2018). An accurate and rapid continuous wavelet dynamic time warping algorithm for end-to-end mapping in ultra-long nanopore sequencing. *Bioinformatics*, *34*(17), i722-i731.
7. Low pass filter python code https://tomroelandts.com/articles/how-to-create-a-simple-low-pass-filter
